# Clinical pregnancy after ICSI: an exploratory transfer-day analysis of female age, AMH, sperm source, and transfer stage

**DOI:** 10.3389/fmed.2026.1823758

**Published:** 2026-06-10

**Authors:** Erhan Hüseyin Cömert, Ümran Karabulut Doğan, Tuğçe Baykara, Mustafa Taş, Yusuf Gençten, Ayşe Ecenaz Yıldırım, Telal Doğruel, Ozan Doğan

**Affiliations:** 1 Private Clinic, Istanbul, Türkiye; 2 Acıbadem Kayseri Hospital, Kayseri, Türkiye; 3 Istanbul Nisantasi University, Istanbul, Türkiye; 4 Afyon Government Hospital, Afyon, Türkiye; 5 Medicalpark Hospital Istanbul, Istanbul, Türkiye; 6 Faculty of Medicine, Istanbul Nisantasi University, Istanbul, Türkiye

**Keywords:** anti-Müllerian hormone, blastocyst transfer, clinical pregnancy, embryo transfer, ICSI, sperm source

## Abstract

**Purpose:**

This study aimed to explore transfer-level factors associated with clinical pregnancy after intracytoplasmic sperm injection (ICSI) using a deliberately lower-claim analytical strategy.

**Methods:**

This retrospective single-centre study screened 332 ICSI-linked records from 2020 to 2025 and analysed 300 embryo transfers from 300 couples. Eligibility criteria, complete-case handling, one-transfer-per-couple preparation, variable coding, logistic regression specification, bootstrap optimism correction, calibration assessment, and software details were documented to support reproducibility. The primary outcome was clinical pregnancy per embryo transfer, defined as an intrauterine gestational sac on transvaginal ultrasonography. Since only 37 events were available, the primary multivariable analysis was restricted to four prespecified candidate variables: female age, anti-Müllerian hormone (AMH), sperm source, and embryo-transfer stage. This restriction was driven primarily by statistical proportionality and the limited event count, rather than an assumption that the omitted variables were not clinically significant. The analysis was intended to examine exploratory associations that could frame cautious transfer-day counselling discussions once all four variables were known; however, it was not designed as a clinical decision support mechanism. Exploratory baseline comparisons, a univariable logistic regression analysis, a four-variable multivariable association analysis, bootstrap-corrected discrimination, descriptive calibration assessment, and a fuller eight-variable sensitivity analysis were used.

**Results:**

Clinical pregnancy occurred in 37 of 300 transfers (12.3%). In the simplified multivariable analysis, older maternal age was associated with lower odds of clinical pregnancy (adjusted odds ratio [aOR] 0.91 per year, 95% confidence interval [CI] 0.83–0.99; *p* = 0.032), whereas higher AMH was associated with higher odds (aOR 2.02 per unit, 95% CI 1.39–2.94; *p* < 0.001). A more invasive sperm-source category was associated with lower odds (aOR 0.39, 95% CI 0.17–0.92; *p* = 0.032), and blastocyst transfer was associated with higher odds than cleavage-stage transfer (aOR 3.90, 95% CI 1.29–11.74; *p* = 0.016). The apparent area under the curve was 0.779, and the bootstrap-corrected area under the curve was 0.750.

**Conclusion:**

In this dataset, female age, AMH, sperm source, and transfer stage showed the clearest exploratory associations with clinical pregnancy per embryo transfer. Since the event count was limited and calibration, coefficient stability, and transportability remain uncertain, the findings should be interpreted as hypothesis-generating. They may support cautious transfer-day counselling discussions but are not sufficient for routine predictive implementation without larger external validation studies.

## Introduction

Transfer-level counselling in assisted reproduction remains challenging, as the information sought by patients during embryo transfer is often more specific than the information provided by broad cycle-level prognosis. Couples commonly ask whether a particular transfer has a realistic likelihood of resulting in clinical pregnancy; however, familiar counselling anchors are still dominated by female age alone. Female age unquestionably remains one of the most reproducible determinants of success across *in vitro* fertilisation (IVF) and ICSI settings; however, transfer-level outcomes are also shaped by ovarian reserve, laboratory selection, and the context of male-factor treatment (1–4).

The majority of established prognostic tools in assisted reproduction were developed for broader cycle-level or cumulative outcomes and were intended primarily to inform expectations over time. Those tools are important; however, they do not always map directly onto the narrower question posed on the day of transfer: What does this specific transfer mean for this couple right now? A transfer-day exploratory framework does not replace cumulative modelling; instead, it asks whether a small set of clinically visible variables can summarise the immediate scenario in a transparent and appropriately cautious way.

An additional reason for focusing on transfer-level analysis is that counselling at transfer occurs after several biologically relevant selection steps have already taken place. By that stage, ovarian response, oocyte maturation, fertilisation, embryo development, and the practical consequences of male-factor management have influenced both embryo availability and the transfer options that remain. An analysis centred on variables visible at or near the transfer decision may therefore be aligned with the real clinical conversation. This does not make transfer-level analyses superior in every setting; however, it does make them relevant to a distinct counselling question.

Among female markers, anti-Müllerian hormone (AMH) is widely used in daily practice because it reflects ovarian reserve and is clinically useful when planning stimulation and anticipating oocyte yield. At the same time, the literature has repeatedly shown that AMH is not a strong stand-alone determinant of downstream pregnancy outcomes. Its value is greater when it is interpreted together with age and treatment-related variables that are more closely related to the actual transfer being evaluated. For that reason, a transfer-level analysis that includes AMH can be clinically significant, with its role framed as complementary rather than deterministic (3–6).

Male-factor infertility is often simplified in prognostic research. Many analyses either omit male variables altogether or reduce them to broad diagnostic categories. In routine ICSI practice, however, the pathway through which sperm is obtained may carry clinically relevant information. Moving from the ejaculated sperm to TESE and then to mTESE usually reflects increasing severity of underlying spermatogenic impairment; therefore, sperm source may function as an accessible procedure-proximal marker of the biological context in which treatment is being performed. This does not mean that the retrieval procedure itself causes poorer outcomes in a simple way; rather, it may summarise a broader severity profile that is otherwise difficult to capture in a compact clinical analysis (7–11).

The embryo-transfer stage also deserves attention in a pragmatic transfer-level framework. Blastocyst transfer may be associated with higher implantation and clinical pregnancy rates in selected populations; however, its interpretation is complex because embryos that reach the blastocyst stage have already survived an important developmental filter. Therefore, the transfer stage is clinically informative while also being partly a marker of prior selection. For counselling purposes, that dual meaning may still be useful as it reflects the actual transfer scenario facing the couple, even if it should not be read as a purely causal treatment effect (12, 13).

The first version of this dataset included a broader set of variables, including metaphase II (MII) oocyte count, azoospermia subtype, male genetic abnormality code, and round spermatid injection (ROSI) use. However, the final analytic cohort contained only 37 clinical pregnancy events. Under those conditions, a broad multivariable specification could give a misleading impression of precision and clinical readiness. The simplified four-variable analysis in the present manuscript should therefore be understood primarily as a consequence of statistical constraint and a lower-claim reporting strategy. Its relative ease of explanation is a practical advantage; however, it is not the primary justification for omitting additional variables.

Accordingly, this revised manuscript has a more restrained objective. We aimed to examine whether female age, AMH, sperm source, and embryo-transfer stage were associated with clinical pregnancy per embryo transfer in a retrospective single-centre ICSI cohort. The analysis is presented as a transfer-day, patient-centred counselling framework rather than as a deployable prediction tool. Any performance summaries are treated as descriptive indicators of how the simplified analysis behaved in this dataset, not as evidence of routine clinical utility.

## Materials and methods

This retrospective observational study was conducted at a tertiary assisted reproduction centre and included ICSI-linked records collected between 2020 and 2025. The unit of analysis was the embryo transfer. Since repeated transfers from the same couple can create within-couple correlation and inflate apparent information if handled naively, one-embryo transfer per couple was retained in the final analytic dataset. The study was therefore analysed as a cross-sectional, transfer-level cohort with 300 independent observations.

### Study population and STROBE flow

**Figure 1:**
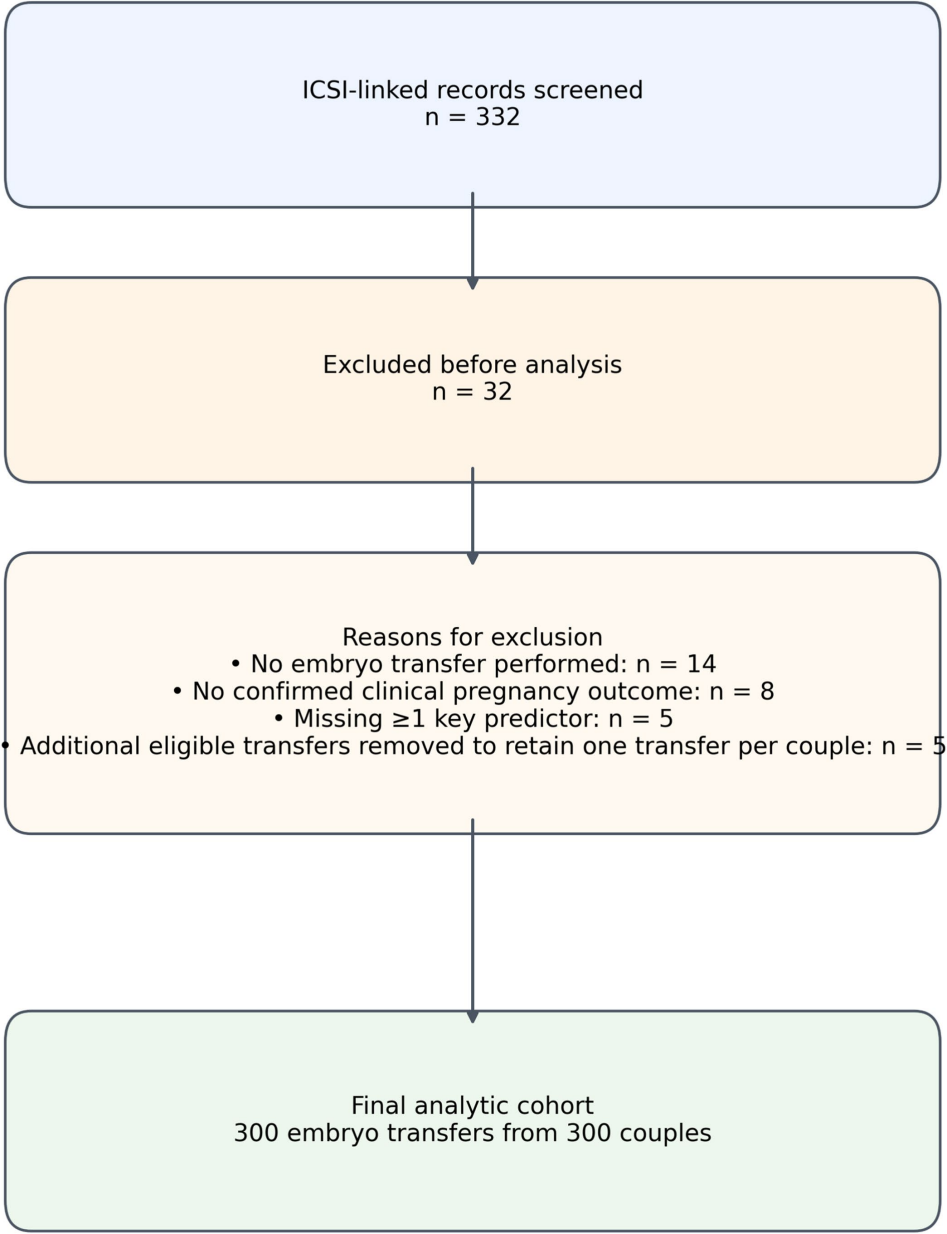
STROBE flow diagram of the analytical cohort.

A STROBE-style screening frame of 332 ICSI-linked records was used for manuscript presentation. Eligibility was assessed at the embryo-transfer level. Records were eligible if they had a documented ICSI procedure, an embryo transfer, an available clinical pregnancy outcome, and complete information for the four main candidate variables: female age, AMH, sperm source, and embryo-transfer stage. Records were not retained if the transfer outcome was not available, if any of the prespecified main variables were missing, or if the record could not be linked reliably to the one-transfer-per-couple analytical structure. After eligibility assessment, exclusion of cases with incomplete variable or outcome data, and retention of only one transfer per couple, 300 embryo transfers from 300 couples remained in the final cohort (Figure 1).

The final cohort was assembled from routinely collected clinical data associated with embryo-transfer outcomes. To avoid within-couple correlation, no couple contributed more than one transfer to the final analysis. When more than one transfer record existed for the same couple in the source screening frame, the analysis-ready dataset retained a single eligible transfer before modelling; the resulting file therefore represented a one-transfer-per-couple complete-case cohort. No additional record-level exclusions or substitutions were made during statistical modelling.

A complete-case approach was used for all primary analyses. No statistical imputation was performed. The denominator for the main descriptive and regression analyses was therefore fixed at 300 embryo transfers, and the number of clinical pregnancy events was fixed at 37. All percentages reported in the tables were calculated using the column-specific denominators, as shown in the table headings.

### Outcome and candidate variables

The primary outcome was clinical pregnancy per embryo transfer, defined as the presence of an intrauterine gestational sac on transvaginal ultrasonography according to institutional follow-up practice. For regression modelling, the outcome was coded as 0 = no clinical pregnancy and 1 = clinical pregnancy. This outcome was chosen because it is clinically recognisable, proximal to transfer-level counselling, and consistently available in the dataset.

The main exploratory analysis included four prespecified candidate variables. Female age was analysed as a continuous variable in years. AMH was analysed as a continuous variable in its original measurement scale. Sperm source was represented using an *a priori* ordered coding of ejaculate = 0, TESE = 1, and mTESE = 2 as a pragmatic gradient of increasing procedural invasiveness and male-factor severity; this coding was intended as a compact clinical summary rather than as proof of equal biological spacing between categories. Embryo-transfer stage was coded as cleavage-stage transfer = 0 and blastocyst-stage transfer = 1.

For the fuller sensitivity analysis, MII oocyte count was analysed as a continuous variable. Azoospermia type and male genetic abnormality code were analysed using the exploratory ordered coding recorded in the dataset. ROSI was analysed as a binary variable, coded as no = 0 and yes = 1. Since several of these additional variables were sparse or clinically heterogeneous, their estimates were interpreted only as exploratory sensitivity findings.

The intended interpretation of this simplified variable set was restricted to counselling immediately before embryo transfer, once all four variables were known. It was not intended for cycle-start prognostication, automated decision-making, or threshold-based treatment selection. The language of the manuscript therefore focuses on exploratory associations rather than clinical prediction.

Additional variables available in the broader dataset were metaphase II (MII) oocyte count, azoospermia type, male genetic abnormality code, and round spermatid injection (ROSI). These variables were included because they were clinically relevant. Rather, they were kept out of the main analysis because the event count was modest and several of these variables were sparse or partly encoded in ordered categories for pragmatic summarisation. They were reintroduced only in a secondary sensitivity analysis to examine whether the central associations for age, AMH, sperm source, and transfer stage were directionally stable.

### Clinical and laboratory context

Ovarian stimulation, oocyte retrieval, fertilisation by ICSI, embryo culture, and embryo transfer were performed as part of routine clinical care according to institutional practice over the study period. This re-analysis did not attempt to model detailed stimulation protocols, embryo grading systems, or laboratory-process variables because these data were not consistently available for a larger specification and because the event count did not support a substantially expanded analysis.

For similar reasons, male-factor characterisation was intentionally simplified. Azoospermia type, male genetic abnormality code, and ROSI were preserved for sensitivity analysis rather than foregrounded in the main analysis. This decision does not imply that those variables are not biologically significant. Instead, it reflects the analytic priority of avoiding overfitting and exaggerated precision in a relatively small number of outcome events.

### Statistical analysis

Continuous variables are presented as mean ± standard deviation, and categorical variables are presented as number (percentage). The baseline characteristics were summarised for the whole cohort and by clinical pregnancy status. Exploratory group comparisons used independent-samples t-tests for continuous variables, and chi-square tests or Fisher’s exact tests were used for categorical variables, depending on cell counts. These comparisons were intended to describe the dataset rather than to serve as a formal variable selection stage. No multiplicity adjustment was applied because the analysis was explicitly exploratory. Several potentially relevant ART descriptors were not available in sufficiently consistent form for a broader descriptive table and are therefore acknowledged in the limitations rather than presented as partially incomplete baseline variables.

The revised analytic strategy was guided by statistical proportionality. With only 37 clinical pregnancy events, a larger main specification could have created unstable coefficient estimates and an overconfident clinical narrative. Therefore, the four-variable analysis was selected as the primary specification. This choice should be read as a limitation-aware response to the available information content of the dataset rather than as evidence that a four-variable analysis is inherently sufficient for clinical care.

Univariable logistic regression models were first fitted separately for each available variable, with clinical pregnancy per embryo transfer as the dependent variable. The main multivariable logistic regression analysis included only the four prespecified variables: female age, AMH, sperm source, and transfer stage. No automated variable selection procedure was used. Age and AMH were retained as linear terms on their original scales for parsimony and readability, while acknowledging the fact that modest non-linearity could not be examined fully in a dataset of this size. Odds ratios were calculated by exponentiating the logistic regression coefficients, and 95% confidence intervals were calculated from the corresponding model-based standard errors.

Effect estimates are reported as odds ratios (ORs) with 95% confidence intervals (CIs). Two-sided *p*-values were reported for descriptive inference, with a p-value of < 0.05 treated as nominally statistically significant but interpreted cautiously because the study was exploratory. Discrimination was assessed using the area under the receiver operating characteristic curve (AUC) calculated from the fitted probabilities. The apparent AUC was calculated in the development dataset.

Optimism-corrected discrimination was estimated using 500 bootstrap resamples. In each bootstrap resample, the four-variable model was refitted, and its performance was compared with performance when applied back to the original dataset. The average optimism estimate was subtracted from the apparent AUC to obtain the bootstrap-corrected AUC. The overall accuracy was summarised using the Brier score.

Calibration was assessed descriptively by comparing observed and estimated clinical pregnancy probabilities across quintiles of estimated risk. Since only 37 events were available and estimated probabilities were concentrated in the lower range, formal calibration modelling was not emphasised. The calibration plot was therefore interpreted as a descriptive check rather than as confirmatory evidence of transportability.

All statistical analyses were performed using Python with standard statistical and machine learning libraries. Decision curve analysis was not presented because no prespecified clinical action threshold was available, and the current study was not designed to support threshold-based decision rules.

#### Ethics

The study protocol was approved by the Acıbadem University and Acıbadem Healthcare Institutions Medical Research Ethics Committee (ATADEK, 2026). Given the retrospective design and the use of anonymised routinely collected data, the requirement for informed consent was waived in accordance with local regulations and the Declaration of Helsinki.

## Results

The final analytic cohort comprised 300 embryo transfers from 300 couples, of which 37 (12.3%) resulted in clinical pregnancy. The mean female age was 31.79 ± 4.25 years, the mean AMH was 1.90 ± 1.03, and the mean MII oocyte count was 10.20 ± 4.32. The majority of transfers involved ejaculated sperm (73.0%), whereas 19.0% used TESE-derived sperm and 8.0% used mTESE-derived sperm. Blastocyst transfer accounted for a minority of transfers overall but contributed a larger share of pregnancies than cleavage-stage transfer.

Table 1 summarises baseline characteristics overall and by outcome group. The clearest difference between transfers that resulted in clinical pregnancy and those that did not result in clinical pregnancy was AMH. The mean AMH was 2.49 ± 0.88 among transfers with clinical pregnancy compared with 1.81 ± 1.03 among transfers without clinical pregnancy. Female age was modestly lower in the pregnancy group, whereas the mean MII oocyte count was similar between the groups.

**Table 1:** Baseline characteristics by clinical pregnancy outcomes.

Variable	Overall (*n* = 300)	No clinical pregnancy (*n* = 263)	Clinical pregnancy (*n* = 37)	*p*
Female age, years	31.79 ± 4.25	31.97 ± 4.22	30.49 ± 4.28	0.054
AMH	1.90 ± 1.03	1.81 ± 1.03	2.49 ± 0.88	<0.001
MII oocytes, *n*	10.20 ± 4.32	10.16 ± 4.28	10.46 ± 4.68	0.717
Azoospermia type				0.571
None	187 (62.3%)	165 (62.7%)	22 (59.5%)	
OA	87 (29.0%)	74 (28.1%)	13 (35.1%)	
NOA	26 (8.7%)	24 (9.1%)	2 (5.4%)	
Sperm source				0.050
Ejaculate	219 (73.0%)	186 (70.7%)	33 (89.2%)	
TESE	57 (19.0%)	55 (20.9%)	2 (5.4%)	
mTESE	24 (8.0%)	22 (8.4%)	2 (5.4%)	
Male genetic abnormality code				0.288
0	242 (80.7%)	209 (79.5%)	33 (89.2%)	
1	41 (13.7%)	39 (14.8%)	2 (5.4%)	
2	17 (5.7%)	15 (5.7%)	2 (5.4%)	
ROSI				0.328
No	237 (79.0%)	205 (77.9%)	32 (86.5%)	
Yes	63 (21.0%)	58 (22.1%)	5 (13.5%)	
Transfer stage				0.014
Cleavage	88 (29.3%)	84 (31.9%)	4 (10.8%)	
Blastocyst	212 (70.7%)	179 (68.1%)	33 (89.2%)	

Continuous variables are shown as mean ± SD. Categorical variables are shown as *n* (%). *p*-values are exploratory and are not adjusted for multiple comparisons.

The categorical distribution of treatment-related variables also suggested clinically plausible patterns. Blastocyst transfer was more frequent among transfers achieving clinical pregnancy than among those that did not. By contrast, the proportion of TESE and mTESE cases was higher in the non-pregnancy group. These descriptive differences are informative for context; however, since the data are observational, they should not be read as direct evidence of causal treatment effects.

Observed event rates reinforce the same exploratory pattern. The clinical pregnancy rate was 15.6% after blastocyst transfer compared with 4.5% after cleavage-stage transfer. Based on sperm source, the corresponding rates were 15.1% for ejaculated sperm, 3.5% for TESE, and 8.3% for mTESE. These crude percentages are useful for clinical intuition, although they do not account for concurrent differences in female age or AMH.

Exploratory univariable logistic regression results are shown in Table 2. Higher AMH was positively associated with clinical pregnancy (OR: 1.93, 95% CI: 1.35–2.75; *p* < 0.001), and blastocyst transfer was also positively associated with clinical pregnancy (OR: 3.87, 95% CI: 1.33–11.28; *p* = 0.013). Female age showed an inverse association at the borderline of conventional statistical significance (OR: 0.92, 95% CI: 0.85–1.00; *p* = 0.049).

**Table 2:** Exploratory univariable logistic regression.

Variable	OR (95% CI)	*p*
Female age (per 1-year increase)	0.92 (0.85–1.00)	0.049
AMH (per 1-unit increase)	1.93 (1.35–2.75)	<0.001
MII oocytes (per 1-oocyte increase)	1.02 (0.94–1.10)	0.696
Azoospermia type (exploratory ordered coding)	0.99 (0.58–1.68)	0.969
Sperm source (ordered coding: ejaculate→TESE→mTESE)	0.47 (0.22–1.03)	0.059
Male genetic abnormality code (exploratory ordered coding)	0.67 (0.31–1.44)	0.304
ROSI (yes vs. no)	0.55 (0.21–1.48)	0.238
Blastocyst vs. cleavage-stage transfer	3.87 (1.33–11.28)	0.013

Each row represents a separate univariable model for clinical pregnancy per embryo transfer.

The univariable association for sperm source was negative and consistent with the final multivariable direction, although the estimate was less precise (OR: 0.47, 95% CI: 0.22–1.03; *p* = 0.059). Other variables available in the broader dataset, including MII oocyte count, azoospermia type, male genetic abnormality code, and ROSI, did not show strong univariable associations in this cohort. This pattern supported using a smaller main specification while still retaining the broader set for sensitivity analysis.

**Figure 2:**
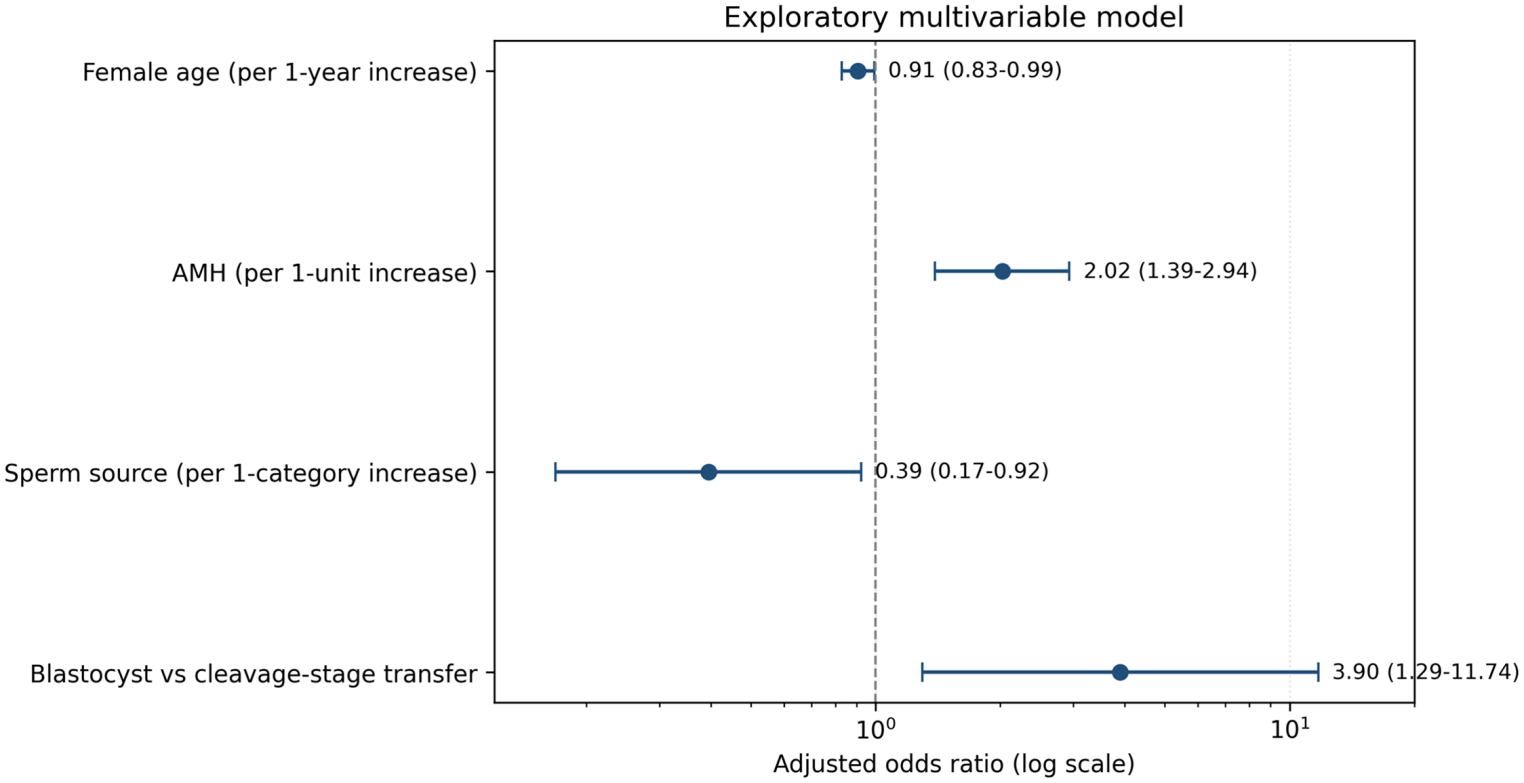
Forest plot of the simplified exploratory multivariable association analysis.

In the simplified four-variable multivariable analysis (Table 3; Figure 2), older female age remained associated with lower odds of clinical pregnancy (adjusted OR: 0.91 per year, 95% CI: 0.83–0.99; *p* = 0.032). Higher AMH remained positively associated with clinical pregnancy (adjusted OR: 2.02 per unit, 95% CI: 1.39–2.94; *p* < 0.001). A one-category increase in sperm source from ejaculate to TESE to mTESE was associated with lower odds of clinical pregnancy (adjusted OR: 0.39, 95% CI: 0.17–0.92; *p* = 0.032). Blastocyst transfer was associated with higher odds of clinical pregnancy than cleavage-stage transfer (adjusted OR: 3.90, 95% CI: 1.29–11.74; *p* = 0.016).

**Table 3:** Simplified exploratory multivariable association analysis.

Variable	Adjusted OR (95% CI)	*p*
Female age (per 1-year increase)	0.91 (0.83–0.99)	0.032
AMH (per 1-unit increase)	2.02 (1.39–2.94)	<0.001
Sperm source (ordered coding: ejaculate→TESE→mTESE)	0.39 (0.17–0.92)	0.032
Blastocyst vs. cleavage-stage transfer	3.90 (1.29–11.74)	0.016

The main analysis was restricted to four variables because only 37 clinical pregnancy events were available in 300 analysed transfers.

The relative size and direction of these associations indicate that the four variables captured three distinct clinical dimensions: female reproductive ageing, ovarian reserve, and the procedural context of both sperm acquisition and embryo transfer. Even so, the coefficients should be interpreted as dataset-specific associations, not as stable, transportable truths. In particular, the sperm-source and transfer-stage estimates are likely to incorporate confounding by indication and selection mechanisms that are only partly captured by the measured covariates.

The broader dataset contained clinically relevant but less frequent or more weakly associated variables. Azoospermia type showed no strong separation between pregnancy and non-pregnancy groups, and the distribution of male genetic abnormality codes was heavily concentrated in the lower categories. ROSI was uncommon. These features reduce effective information for multivariable estimation even when the nominal sample size is 300. In practical terms, they help explain why a broader specification can look more comprehensive on paper while adding relatively less stable signal in the present dataset.

**Figure 3:**
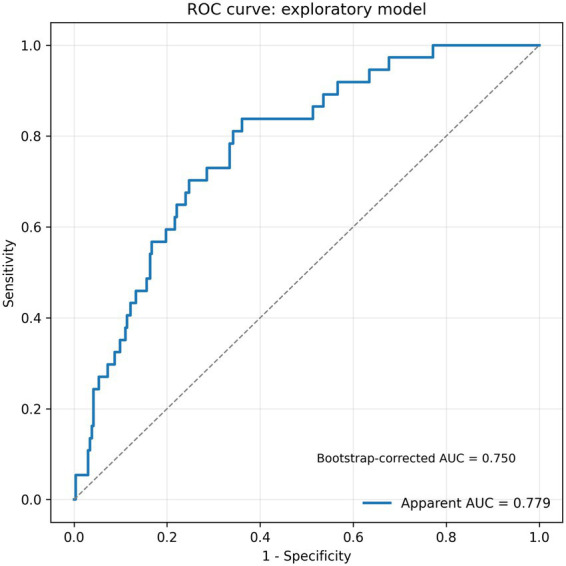
Receiver operating characteristic curve of the simplified exploratory analysis.

**Figure 4:**
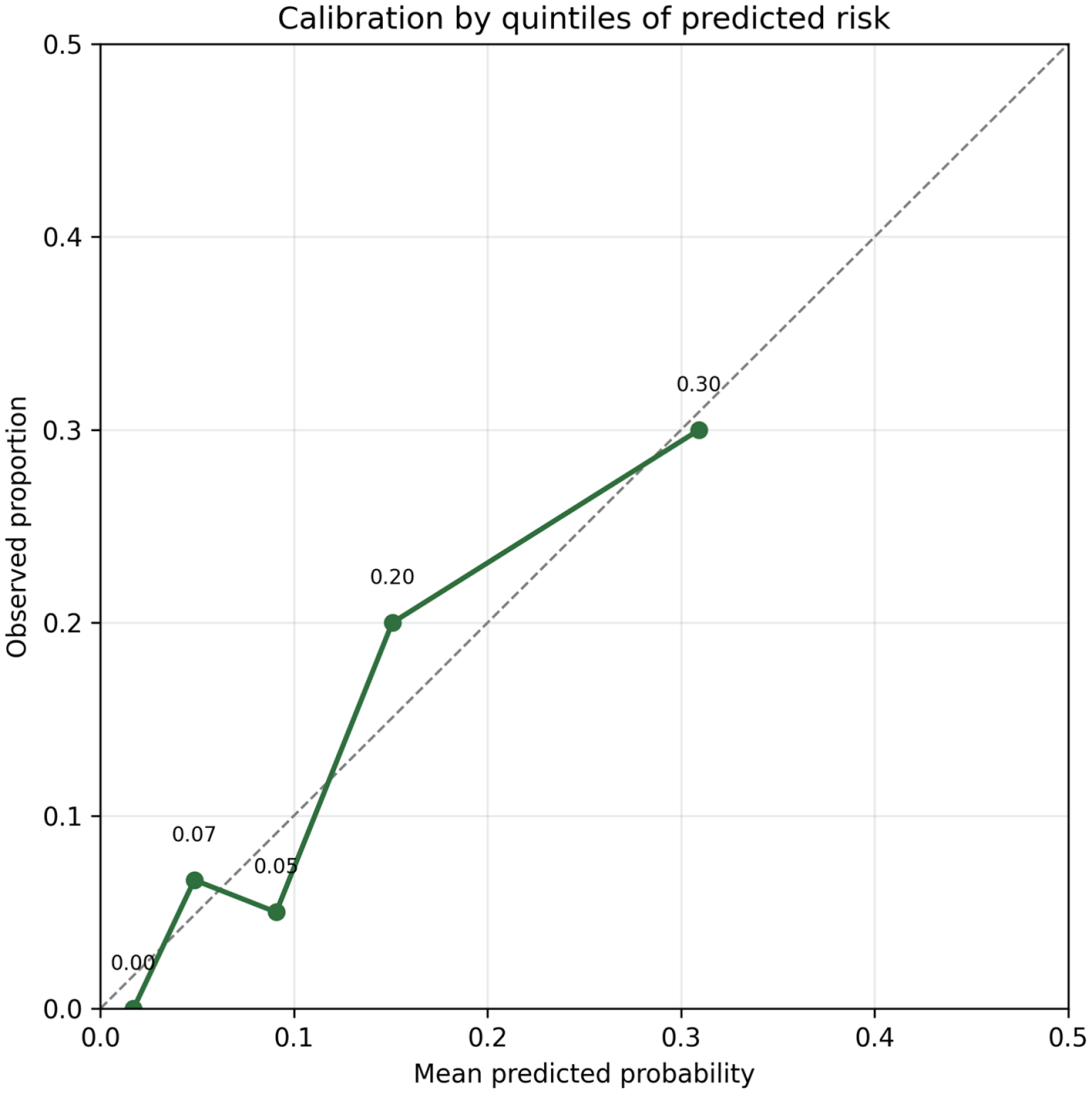
Calibration plot by quintiles of estimated risk.

Descriptive performance was moderate and should be interpreted cautiously. The apparent AUC was 0.779, the bootstrap-corrected AUC was 0.750, and the Brier score was 0.097 (Figures 3, 4). The gap between apparent and optimism-corrected discrimination was modest, but the limited number of events precludes strong claims about robustness. Calibration was explored visually only; because estimated probabilities were concentrated in the lower range and higher-risk strata contained few observations, the calibration display should be read as descriptive rather than confirmatory.

The fuller eight-variable sensitivity analysis showed similar directions and approximate magnitudes for the four main variables, while the apparent AUC increased marginally to 0.791 (Table 4). As shown in Table 4, the central female and procedural signals remained recognisable: older age continued to show an inverse association, AMH retained a positive association, sperm source remained negative, and blastocyst transfer remained positive. By contrast, MII oocyte count, azoospermia type, ROSI, and male genetic abnormality code did not contribute strong independent associations in the same specification. Since the fuller analysis included sparse additional variables and exploratory ordered coding for some male-factor measures, it should be interpreted as a robustness check rather than as a preferred clinical specification.

**Table 4:** Sensitivity analysis using the fuller original variable set.

Variable	Adjusted OR (95% CI)	*p*
Female age (per 1-year increase)	0.90 (0.82–0.99)	0.024
AMH (per 1-unit increase)	2.14 (1.45–3.15)	<0.001
MII oocytes (per 1-oocyte increase)	1.00 (0.92–1.09)	0.985
Azoospermia type (exploratory ordered coding)	0.87 (0.49–1.55)	0.638
Sperm source (ordered coding: ejaculate→TESE→mTESE)	0.38 (0.16–0.90)	0.028
Male genetic abnormality code (exploratory ordered coding)	0.53 (0.24–1.19)	0.126
ROSI (yes vs. no)	0.62 (0.21–1.77)	0.370
Blastocyst vs. cleavage-stage transfer	3.80 (1.25–11.57)	0.019

Sensitivity analysis includes age, AMH, MII oocytes, azoospermia type, sperm source, male genetic code, ROSI, and transfer stage.

## Discussion

This revised manuscript deliberately makes a narrower claim than the original broader version. The principal finding is that, within this retrospective single-centre dataset, female age, AMH, sperm source, and transfer stage carried the clearest exploratory signal for clinical pregnancy per embryo transfer after ICSI. This study should therefore be read as a transfer-day patient-centred counselling analysis rather than as a validated clinical prediction tool.

The age and AMH findings fit well with the broader assisted reproduction literature. Female age remains the most stable marker of reproductive potential because it captures biologically important changes in oocyte competence and embryonic aneuploidy risk. AMH, in contrast, is best understood as a reserve marker whose relationship with downstream pregnancy is context-dependent. Our results are consistent with the literature in showing that AMH can add useful information when interpreted together with age and transfer-related variables, even though it should not be presented as a stand-alone determinant of success (1–6).

The observed association between blastocyst transfer and higher clinical pregnancy probability should be interpreted carefully. On the one hand, this finding is directionally concordant with prior comparative research that suggests improved transfer efficiency in selected circumstances (12, 13). On the other hand, embryos that reach the blastocyst stage have already passed a strong developmental selection step. The association therefore reflects both transfer-stage choice and embryo selection. For clinical counselling, this combined signal may still be useful because it reflects the real scenario presented to the patient at transfer. For causal inference, however, the estimate is clearly vulnerable to selection effects.

Sperm source deserves equally cautious interpretation. In many clinical settings, escalation from ejaculated sperm to TESE or mTESE reflects increasing severity of male-factor infertility. The negative association observed in this study should not be simplified into a claim that surgical retrieval inherently reduces pregnancy probability. More plausibly, sperm source acts as a compact marker of severity, prior failed pathways, and unmeasured biological characteristics, which is why it may be clinically informative in an exploratory counselling analysis: It can proxy a complex male-factor context that is otherwise difficult to summarise using a few variables (7–11).

One of the main methodological decisions in this revision was to foreground the limited event count rather than present simplicity as a stand-alone strength. With only 37 clinical pregnancy events, a broader eight-variable main specification risked overstating what the data could support. The four-variable analysis should therefore be interpreted as a statistically constrained, lower-claim specification. Its bedside intelligibility is useful; however, it should not obscure the fact that important variables were omitted because the dataset could not support a more elaborate and stable multivariable analysis.

The sensitivity analysis helps contextualise this decision. The central associations remained directionally similar when the broader variable set was added, and the apparent AUC increased only marginally. This does not prove that the simplified analysis is sufficient; rather, it suggests that, in this dataset, the additional variables did not add a clearly stable independent signal. Some overfitting and coefficient instability cannot be excluded, even with prespecification and bootstrap correction. The sensitivity analysis should therefore be read as a robustness check, not as validation.

Another reason for restraint is that apparent discrimination can easily be over-interpreted in small retrospective datasets. An AUC in the mid-0.70 range may look encouraging; however, the practical meaning of that number depends on calibration, generalisability, and the stability of the underlying coefficients. An analysis can separate outcomes reasonably well inside the development sample and still perform poorly when transported to a different centre with different laboratory thresholds, patient selection, and sperm retrieval practices. For that reason, this study treats AUC, Brier score, and calibration as descriptive diagnostics rather than as badges of clinical readiness.

The same principle applies to *p*-values and confidence intervals. Although some estimates in this analysis met conventional thresholds for statistical significance, this does not convert observational associations into simple causal effects. For example, the association between blastocyst transfer and clinical pregnancy almost certainly mixes biological competence, laboratory selection, and treatment choice. Similarly, the association with sperm source likely reflects clinical severity context rather than a direct effect of the retrieval pathway itself. The confidence intervals are informative about precision within this dataset, but they do not resolve residual confounding or between-centre heterogeneity.

This analysis has several practical strengths. First, it focuses on variables that are clinically recognisable and available close to the time of transfer, which increases relevance to the counselling moment. Second, it makes the analytical trade-off explicit: lower ambition in exchange for greater transparency and less risk of overclaiming. Third, this manuscript reports not only coefficient estimates but also descriptive performance measures, calibration, and a sensitivity analysis, allowing readers to see how the analysis behaves beyond a single headline AUC.

A related practical point is that counselling-oriented analyses serve a different purpose from mechanistic or aetiological studies. A transfer-day counselling framework does not need to encode every biologically interesting pathway to be useful; it needs to organise information that clinicians and couples can discuss at a meaningful decision point. In that sense, age, AMH, sperm source, and transfer stage are attractive not because they are exhaustive; however, they are readily interpretable and jointly represent the female, male, and embryology context of a transfer. The trade-off is that interpretability should never be mistaken for completeness. Important unmeasured factors almost certainly remain outside the analysis, including laboratory-specific embryo selection practices, stimulation nuances, endometrial characteristics, and subtle male-factor biology.

Important limitations remain. The study was retrospective and single-centre; therefore, confounding, selection bias, and centre-specific practice patterns may influence the observed associations. The number of events was modest, which limits coefficient stability and prevents any strong conclusion about transportability. Several potentially relevant ART variables, including finer embryo-quality measures, endometrial or protocol-related details, and other laboratory-process variables, were not modelled consistently enough for inclusion in this framework. In addition, possible non-linearity in continuous variables and richer category-specific modelling of sparse male-factor variables could not be examined reliably in this dataset. Finally, external validation was not available, and, therefore, even moderate apparent performance should not be interpreted as readiness for clinical deployment.

These limitations have a direct impact on interpretation. This analysis can support cautious conversation with couples by indicating that, in this dataset, younger age, higher AMH, ejaculated sperm, and blastocyst transfer were each associated with higher observed clinical pregnancy after transfer. What it cannot do is provide a definitive personalised probability that should independently guide treatment decisions based on clinician judgement and local laboratory context. The appropriate use of these results is therefore explanatory and hypothesis-generating rather than prescriptive.

The choice of clinical pregnancy per embryo transfer as the primary outcome also deserves comment. Live birth is often the preferred endpoint in reproductive medicine; however, clinical pregnancy is still a meaningful and commonly reported intermediate outcome, particularly in retrospective datasets where follow-up completeness may differ across downstream endpoints. In this study, clinical pregnancy was the most consistently available transfer-level outcome and aligned closely with the counselling question embedded in the dataset. Even so, future validation work should examine whether the same simplified variable set remains informative when live birth or cumulative outcomes are considered.

In broader terms, this study illustrates an important reporting principle in reproductive prediction research. Not every dataset justifies a full-scale prognostic tool, and not every statistically significant association should be translated into a strong clinical claim. In some situations, the most responsible approach is to simplify the analysis, narrow the language, and show clearly what remains uncertain. That was the intention of the present revision, and it is why the manuscript emphasises exploratory interpretation throughout.

From a practical standpoint, this manuscript supports a modest counselling message rather than a rule. When discussing prognosis with a couple at transfer, clinicians may reasonably recognise that female age and AMH continue to be essential, that sperm source may summarise important male-factor context, and that embryo-transfer stage carries information about the developmental path already traversed by the embryo. What clinicians should not do is convert these coefficients into a false sense of certainty. The estimates were derived from one centre, one retrospective dataset, and some events; they are best used to frame discussion, not to replace judgement.

Future research should proceed in two steps. First, the simplified four-variable framework should be tested in an external dataset from one or more centres that differ in patient mix and laboratory practice. Second, if the core associations persist, a prospective study could evaluate whether communicating a restrained transfer-level probability estimate meaningfully improves counselling quality or decision satisfaction and could predefine the clinical thresholds needed for interpretable decision curve analysis. Until then, this analysis is more useful for prioritising candidate variables than for formal clinical implementation.

## Conclusion

In this single-centre retrospective ICSI cohort, older female age and more invasive sperm-source categories were associated with lower odds of clinical pregnancy per embryo transfer, whereas higher AMH and blastocyst transfer were associated with higher odds. These associations were directionally stable in a fuller sensitivity analysis, but the overall work remains exploratory because of the modest event count, retrospective design, limited calibration certainty, and lack of external validation. These findings may inform cautious transfer-day couple counselling and may help prioritise variables for future research, but they are not sufficient for routine predictive implementation without larger multi-centre validation studies.

## Data Availability

The de-identified data supporting the findings of this study are available from the corresponding author upon reasonable request, subject to institutional approval and applicable data protection regulations. The data are not publicly available because they contain participant-level clinical information.
